# S10-2 Instruments for the analysis of national-level physical activity and sedentary behaviour policies: a systematic review

**DOI:** 10.1093/eurpub/ckac093.051

**Published:** 2022-08-29

**Authors:** Željko Pedišić

**Affiliations:** Institute for Health and Sport, Victoria University, Melbourne, Australia

**Keywords:** physical activity, sedentary behaviour, national policy, instruments

## Abstract

**Background:**

Researchers and policy makers have developed and used a variety of instruments to assess physical activity policies. Data on the available instruments and their properties have never been summarised in a systematic way. In this review we, therefore, aimed to identify and critically assess available instruments for the analysis of national-level physical activity and sedentary behaviour policies and provide recommendations for their future use.

**Methods:**

A systematic search of grey and academic literature was conducted in Scopus, SPORTDiscus, PubMed/MEDLINE, Web of Science, Networked Digital Library of Theses and Dissertations, and Open Access Theses and Dissertations, on the websites of three large international organisations for physical activity promotion, and using Google. The Comprehensive Analysis of Policy on Physical Activity (CAPPA) framework was used to assess and classify the instruments, based on their purpose, the policy sectors and scope of analysis they cover, and the policy types and stages of policy cycle they inquire about.

**Results:**

Among a total of 22,071 screened references, we found 26 publications describing 16 instruments that met the inclusion criteria. All the available instruments are intended for analysing some aspects of physical activity policy. Only two instruments include questions about sedentary behaviour policy. None of the instruments can be used to analyse all the relevant policy components. Only a few instruments refer to the agenda-setting and endorsement/legitimisation stages, the effects of policy, and the research and tourism sectors. The termination and succession stages of the policy cycle and unwritten formal statements and informal policies were not addressed by any of the available instruments.

**Conclusion:**

Our findings indicated there is a need to design new instruments or adapt the existing ones to facilitate a more comprehensive analysis of national physical activity and sedentary behaviour policy. It would be extremely time-consuming to analyse all important components of physical activity and sedentary behaviour policy in a single analysis; hence the development of complementary instruments, where each one of them inquires about a specific policy component, might be a way forward.

